# The effect of three-circle post standing (Zhanzhuang) qigong on the physical and psychological well-being of college students: A randomized controlled trial

**DOI:** 10.1097/MD.0000000000026368

**Published:** 2021-06-18

**Authors:** Jiaxuan Lyu, Yulong Wei, Hangyu Li, Jingjing Dong, Xinzheng Zhang

**Affiliations:** Department of Acupuncture-Moxibustion and Tuina, Beijing University of Chinese Medicine, Beijing, China.

**Keywords:** complex algorithm, electroencephalogram, heart rate variability, randomized controlled trial, three-circle post standing qigong

## Abstract

**Background:**

Qigong has a long-term application by integration of mind, breath and body to prevent and cure diseases. Researches show that qigong practice could adjust anxiety, the mechanism may found on brain and heart functions. Currently there are limitations on qigong's anxiety-release mechanism study between mind and body, and existing studies lack of evidence on electrophysiology research. Our objective to analyse qigong's anxiety-release effect and mechanism.

**Methods:**

A two-arm randomized clinical trial with 144 qigong naïve anxiety subjects without cerebral or cardiovascular diseases or other severe syndromes will be allocated to either a body and breath regulation group (n = 72) or a body regulation group (n = 72). Participants will conduct three-circle post standing qigong exercise 5 times per week for 8 weeks, while the three-circle post standing qigong combined with abdominal breath regulation (TCPSQ-BR) group will combined with abdominal breath regulation. The primary outcome will be the Self-Rating Anxiety Scale (SAS), and the secondary outcome will be complexity-based measures of heart rate and electroencephalogram (EEG) signals assessed at baseline and 8 weeks. Multiscale entropy analysis will be used as measure of complexity.

**Conclusion:**

This study will be investigate the effects of qigong's anxiety-release by SAS, and will analyze the coordinates of EEG and heart rate variability (HRV) signals before and after three-circle post standing qigong (TCPSQ) practice, and to analyse their synergies by complex signal process method.

**Ethics and trail registration:**

The protocol was approved by the institutional review boards of Beijing University of Chinese Medicine (2018BZHYLL0109). This study was registered with the “Chinese Clinical Trail Registry” in the WHO Registry Network (ChiCTR-Bon-17010840).

## 1 Introduction

With the epidemic of COVID-19, quarantine life increases stress, and somatic symptoms are prevalent lately,^[1]^ and emotion-related disturbances, such as depression and anxiety, have been linked to electroencephalogram (EEG) asymmetry.^[2,3]^ Recently, large prospective epidemiologic studies have established a connection between cardiovascular disease and several psychological conditions, including depression, chronic psychological stress, and anxiety.^[4]^ The mechanism by which anxiety disorders contribute to these symptoms is highly associated with mind-body interactions. Our body is a complex unit with connections between 2 systems, 2 tissues, and 2 cells. Their dynamic functions comprise the biological system. Malfunction of any part would be a threat to our health, and so are the mind and body. Clinically, we found that mental and physical symptoms always appear together and affect each other, which would have an impact on the diagnoses and prognoses. However, it is not clear how they connect and how their functions affect each other. It is difficult to observe this connection in a healthy system, so we will study subjects with anxiety. As a mindful exercise, qigong could provide a unique vision for it could make adjustments to both mind and body simultaneously.

The Chinese medical qigong (CMQ) is a component intervention with complex effects, and research suggests that it affects physical and mental dynamics. Regulation of the mind and body could act as a complementary prescription for chronic disease rehabilitation, such as cancer recovery,^[5–9]^ poststroke recovery,^[10–14]^ and postoperative recovery,^[15,16]^ to relieve pain and restore body function. Another common application is the use of CMQ as an alternative therapy. In addition to the supportive function of clinical conditions, as a preventative and rehabilitative intervention, CMQ can also be used to improve and maintain health; for example, controlling blood pressure^[17–27]^ and glycemia,^[28–31]^ could help build a healthy lifestyle.

There are various types of qigong forms. Since the standing position has the advantage of stretching our muscles, it could help with flows of qi and blood. Here, we choose the three-circle post standing qigong (TCPSQ), which is a representative static exercise that can be traced back to martial arts, is a sample that is easy to operate for qigong naïve. TCPSQ has the characteristics of bilateral symmetrical physical body posture, the balance between the left and right sides of the body, and between the front and back sides of the body is significant. Combined with breath regulation connecting the inner and outer body, the mind and body can be intentionally linked during practice, so the integration of three adjustments can be achieved.

EEG is a nonlinear complex physiological signal with multiple temporal scales, and among modern neuroimaging modalities, EEG has drawn extensive attention to investigate neuronal brain functions and dynamics because of its features of being convenient, noninvasive, and inexpensive.^[32]^ Since studies have shown that EEG can provide functional brain network activities to interpret emotional information, EEG analysis based on a complex network of graph theory has been widely used in clinical researches.^[33,34]^ With an intentional qigong prictice, EEG signals can show changes in one's mind function and brain dynamics.

Heart rate variability (HRV) is the variation in the time intervals of adjacent heartbeats and can be used to quantify autonomic activities.^[31,32]^ Heart rate rhythms exhibit features of complex systems that reflect dynamic, nonstationary, and nonlinear properties. HRV can be associated with exercise capacity; as a noninvasive technique, spectral analysis of HRV has been used widely for examining outflows of the heart, which represent the function of the sympathetic and parasympathetic nervous systems, and can also be associated with the presence and prognosis of cardiac disorders. Therefore, we could investigate and predict adults’ exercise capacity and emotional disorders with cardiac function using HRV. Since the autonomic nervous system cannot be deliberately controlled, it can be adjusted by breathing control. During qigong practice, the mind, body, and breath can be regulated at the same time, which is a good opportunity to learn how intentionally breath regulation affects the autonomic nervous system and get to know the mechanism of their coordination.

Our primary outcome will be the Self-Rating Anxiety Scale (SAS) score, which is an intuitive result of qigong reducing anxiety disorder syndromes. The secondary signals are EEG and HRV; 2 electrophysiological signals represent functions of the central and autonomic nervous systems, which are affected by anxiety syndromes. Both the EEG and HRV are nonlinear and nonstationary signals. Traditional linear analysis methods may cause message loss when processing complex signals. In this study, we re-investigate the features of EEG and electrocardiogram (ECG) signals, and introduce multiscale entropy (MSE), a widely used metric of complexity measure for physiologic signals,^[35–38]^ proposed as a measure of the complexity of a physiologic time series,^[39]^ to estimate the nonlinear complexity of across EEG and the dynamic properties of HRV based on 2 innovative analysis algorithms,^[40–47]^ which could discern features such as spectral and temporal features extracted from the EEG and ECG signals.^[32–45]^

## 2 Methods/designs

### 2.1 Study design

**Figure 1:**
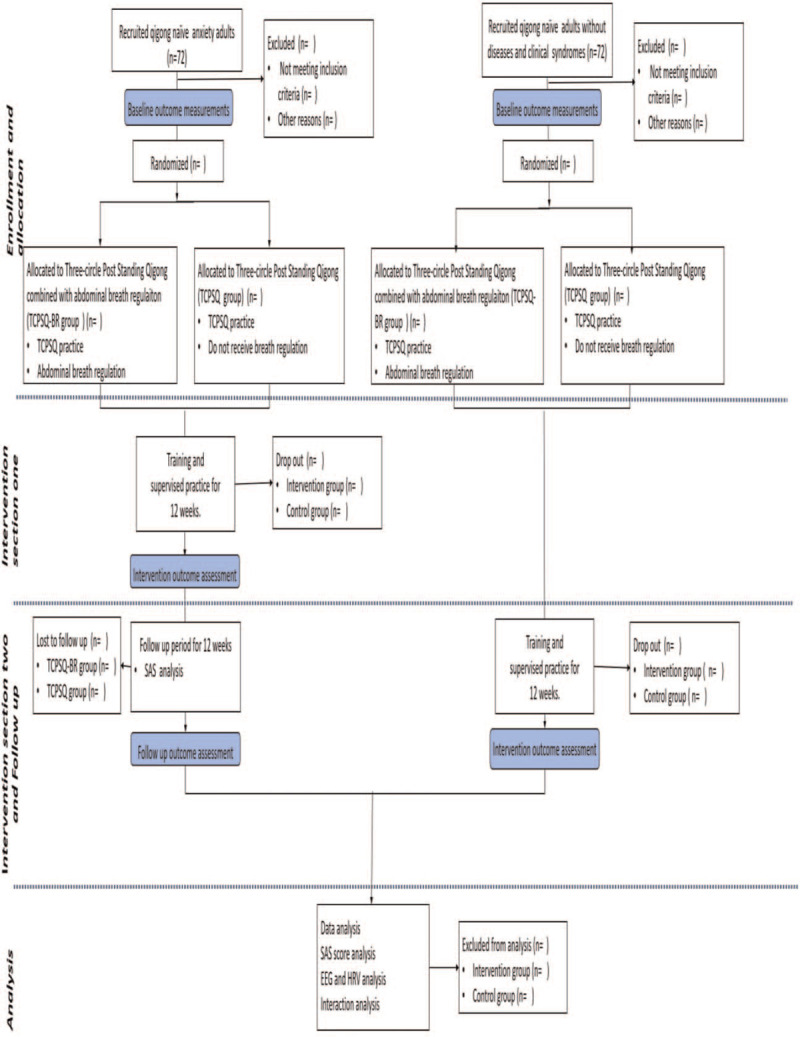
Flowchart of the study design.

This study was a two-section, two-arm, randomized controlled trial. The first section compares the anxiety release effect between three-circle post standing qigong combined with abdominal breath regulation group and three-circle post standing qigong group for pre- and parallel comparison between groups. The second section has 2 targets: the first is to make a follow-up assessment on the anxiety release effects, and the secondary target is to research the mechanism of mind body coordination. The following outcomes were measured: EEG and electrocardiography (ECG). The study design is illustrated in Figure 1.

### 2.2 Ethics

The protocol was approved by the institutional review board of Beijing University of Chinese Medicine, where the study will take place. This study was registered with the

“Chinese Clinical Trail Registry,” Republic of China, which is a registry in the WHO Registry Network. Register number: ChiCTR-Bon-17010840. Registered on October 12, 2018, http://www.chictr.org.cn/showproj.aspx?proj=18439.

This study will be monitored by an independent data and safety monitoring board. Advertisements for voluntary subjects will be published online. All participants provided written informed consent.

### 2.3 Participants

#### 2.3.1 Inclusion criteria

The inclusion criteria are as follows:

1. Volunteers age between 18 and 25 years,^[46]^ right-handed;
2. Self-Rating Anxiety Scale (SAS) scores over 50;^[47]^
3. Fluency in mandarin and signed Informed consent;
4. Finish all training and test process.

### 2.4 Exclusion criteria

The exclusion criteria are as follows:

1. Qigong master or with experience of qigong practice;
2. Severe mental illness;
3. History or Family History of mental illness;
4. Nervous system, respiratory system, cardiovascular and cerebrovascular diseases or taking therapeutic drugs;
5. Malignant tumors or other serious chronic diseases; infectious disease; or cardiovascular, liver, kidney, gastrointestinal, and blood system diseases;
6. Scalp trauma or Cerebral insufficiency;
7. Epilepsy or history of epilepsy;
8. The presence of metal or cardiac pacemaker implants;
9. Musculoskeletal system diseases or other sports contraindications;
10. Chronic cardiovascular or cerebrovascular diseases such as hypertension and diabetes;
11. Prior or current participation in a clinical trial influenced the evaluation of the study results within the 8-week period.

### 2.5 Withdrawal criteria

Participants will be withdrawn from the trial if they present any of the following conditions:

1. Poor compliance or noncompliance (assessed by mean compliance <85% at the last estimation)
2. Occurrence of serious adverse events, such as inability to progress because of sudden disease
3. Subjects required to exit at any time;
4. Members in the TCPSQ group regularly engaged in TCPSQ or other kinds of mindful exercises.
5. Subjects with severe bio-electrical signal loss or damage will be withdrawn from the group during processing.

### 2.6 Participant recruitment

We will recruit 144 participants and provide written consent to all participants before enrolment in the study.

The clinical research coordinator (CRC) will initially screen each potential participant against the inclusion and exclusion criteria by telephone. Willing potential participants will be given an appointment at the center of the qigong trial in Beijing University of Chinese Medicine. A screening log was maintained to record the inclusion/exclusion criteria for all participants. The CRC will briefly explain the purpose of the study and ask if they have an interest in participating. The CRC will also record basic demographic and medical information.

### 2.7 Sample size

No previous clinical trials have evaluated the effect of CMQ on the interaction between the mind and body. Therefore, this research was designed to follow the SAS study of CMQ to calculate the appropriate sample size for future randomized clinical trials. The mean power of the CMQ treatment group was 53.77, with a standard deviation of 7.3, while the TCPSQ group had a mean SAS score of 55.29. And standard deviation of 10.13. According to the aforementioned results, we calculated the sample size using PASS 11.0 (NCSS, LLC. Kaysville, UT, USA), set alpha as 0.05, and set the test effectiveness is 0.90. As a result, we will include 30 participants in each group, considering the 20% withdrawal rate; we will include 36 subjects for each group, which contains a total sample size of 72 subjects for evaluating the effect of qigong's anxiety release effect.^[48–51]^ The second section will analyze the mind–body interaction, and no previous research can be found, so we will include healthy subjects without any basic diseases or symptoms matching the subjects with anxiety groups; the second section will have a sample size of 72. With a total sample size of 144.

### 2.8 Randomization and allocation concealment

Both the anxiety and healthy subjects admitted to the study will be randomized to either the three-circle post standing qigong combined with abdominal breath regulation (TCPSQ-BR) group or the TCPSQ group. Participants will be randomized before the first treatment using an online centralized randomization service. Randomization will be stratified according to age and sex.

Allocation concealment will be ensured, as the service will not release the randomization result until the patients are recruited into the trial after all baseline measurements are completed.

### 2.9 Informed consent

Prior to the study, the general process of the study and the responsibilities of participants and researchers will be explained to all potential subjects. We will confirm that they volunteered to join the trial and told them that they were free to leave if they wanted to. Written informed consent was obtained from each participant before the start of any related interventions. A research assistant will be responsible for obtaining informed consent from all the participants.

### 2.10 Collection of demographic information

Each subject's medical history, including current medication, surgical history, and the presence of other diseases, will be recorded at baseline. To investigate other risk factors for cardiovascular or cerebrovascular diseases, lifestyle factors, including diet, exercise, smoking, and alcohol consumption, will also be documented. Height, weight, age, and other demographic information were also measured.

### 2.11 Blinding

It will be difficult to blind the participants and qigong coaches because the TCPSQ group will not receive any breath regulation. Therefore, we make strict rules for each investigator to define their obligation, recruitment, allocation, qigong treatment, data collection, and analyses will be assigned to different researchers. The allocation sequence and blind code will be preserved by an independent project manager who is irrelevant to all other trail segments, and will be released until the statistical analysis is completed. The project manager and qigong coaches cannot take part in the assessment of outcome; the outcome assessors, laboratory technicians, data managers, and the statistical analyzer will not be involved in the participants’ screening and allocation. Outcome assessors will be blinded to group allocation and will not be involved in providing interventions.

### 2.12 Intervention

Although there have been several CMQ trials, the type of CMQ used in these trials was diverse. We have developed a TCPSQ protocol for the research of psychosomatic synergy based on the training scheme originating from the TCPSQ recorded in Traditional Chinese Medicine Qigong^[52]^ (“13th Five-Year” planning teaching materials of China National Higher Education of Chinese Medicine, Beijing: China Press of Traditional Chinese Medicine. Editor: Tianjin Liu, Wenchun Zhang) and Chinese Medicine Qigong Training Guidance^[53]^ (Beijing: China Press of Traditional Chinese Medicine. Editor: Yulong Wei Two qualified coaches with at least 10 years of experience in CMQ education will participate in the TCPSQ intervention period.

A TCPSQ will be taught by an instructor with expertise in qigong. The participants will visit the clinic and take at least 5 qigong classes per week for 12 weeks. Each qigong class lasted 50 minutes. The purpose of qigong exercise is to familiarize themselves with the TCPSQ practice to integrate mind, body, and breath regulations into 1.

The compliance of the subjects will be assessed in terms of the number of qigong training and practice attended and the feedback from each subject by questionnaires. Every possible effort is made to keep subjects actively engaged in the protocol following attendance and practice guidelines. No medications that affect the cardiovascular or cerebrovascular system were permitted during the study.

### 2.13 Intervention regimen

TCPSQ training will consist of three consecutive half-days, while abdominal breath regulation will take another 3 consecutive half-day initial workshops conducted by 2 qualified coaches, a 12 weeks period of TCPSQ practice under supervised coaches, and a 12-week follow-up.

During the initial workshop, participants will receive a training process, which consists of instructions about the source and characteristics of TCPSQ, get to know its essence and benefits, and the relevant traditional Chinese philosophy and Chinese medicine knowledge relate to TCPSQ, to help them understand and perform TCPSQ. The instruction of the TCPSQ will be both verbal and visual. Participants will also be introduced into the study procedure and precautions. Participants will also be required to learn key movements known as “regulating body, breathe, mind” and ancillary exercises based on multiple repetitions of the TCPSQ until they acquired. This process was confirmed by professional coaches. The coaches will be required to supervise each individual to ensure that the movements are practiced correctly.

Once the training process is completed, participants will be asked to practice the TCPSQ under supervision. Subjects in the TCPSQ and TCPSQ-BR groups will undergo the 12 weeks period of regular practice together with a frequency of 5 days per week. Practice will be performed for 50 minutes per day, each session will include a 10 minutes warm-up, a 30 minutes TCPSQ practice, and a cooling down portion for 10 minutes. (Table 1). The instructors will supervise each individual to ensure that the movements are practiced correctly.

**Table 1:** Composition of three circle standing qigong.

Stage	Motions
Warm-up	Normal state
1st	Baseline, steady standing with eyes closed
2nd	Separate two feet
3rd	Toes slightly point inside, there is a circle of arches between two feet
4th	Two arms hold a circle
5th	Hold a circle between two hands
6th	Close eyes and take deep, slow and even abdomen breath
7th	Take 3 deep breath and open your eyes
Cool-down	Go back to normal state

All participants will be required to complete a self-made TCPSQ practice self-evaluation scale (including practice difficulty, feelings of relaxation, pain and fatigue, sweat, saliva secretion, warmth of body, breath control, meditation, and physical and mental reactions during and after the training) and record their daily practice reports during the intervention period.

To eliminate the bias of participants’ extra activities, participants in both groups were asked to maintain a diet, including the type and intensity of physical activity or exercise, as well as daily sedentary time and sleep duration throughout the study.

### 2.14 TCPSQ group

The TCPSQ group will receive only TCPSQ practice instruction when the TCPSQ-BR group practiced TCPSQ combined with abdominal breath regulation, and did not know any details about qigong practice. They will be asked not to participate in any kind of breath regulation, such as yoga or other forms of qigong.

### 2.15 Follow-up period

All participants in Study section 1 returned to their original lifestyles during the 12-week unsupervised follow-up period. Participants will be required to record their daily physical activities and sports information. Records will be collected by researchers for following up every day by email or phone calls.

The SAS will be re-evaluated at the end of the follow-up period. The follow-up assessment was designed to evaluate the long-term effects of TCPSQ on anxiety subjects’ physical and psychological health.

The second section of study won not have follow-up period.

### 2.16 Quality control

1. Quality control for Researchers:
  We trained our researchers before the study began. All researchers will be asked to memorize the quality and compliance monitoring processes, make sure they are familiar with all study designs, and follow the instructions; we will assign the recruitment, allocation, coaches, data collection, and analyses work to a specific person, define their obligations, and strictly prohibit any disclosure between researchers. All sealed information will be preserved by the representative researchers until the trial is completed.
  We will make assessments of coaches’ teaching skills before the training process, to eliminate the differences in teaching methods between coaches, so that all subjects could receive the same classes from the 2 coaches, the individual-caused difference can be minimized. All researchers will also be trained to unify their intervention or test skill at every stage of the study when they face participants or communicate with them to avoid personal interference bias.
2. Quality control of participants
  During the training and supervised practice phase, the researchers will check in all participants and summarize them after each training; they will contact the subjects who did not attend the training, get to know the reasons why they did not show up, and urged them to finish their training; after each training, the researchers asked the subjects about their feelings of practice, to scale the quality by their feedback, record their problems on the report forms, and deal with the problems they may have.
  When collect the bioelectronic signals, to avoid the possible interrupt or forgotten of practice for the participants who get later test because of the tests order, the participants will be asked to continue their practice until the end of the individual test.
  All participants were required not to share their breath regulation details with the members of the TCPSQ group, and members of the TCPSQ group should not practice any form of breath regulation during the 24 weeks to act as a parallel control.

### 2.17 Outcome measures

The primary outcome was the SAS score. The scales will be collected before (visit 1) and after qigong exercises (visit 2), and after the follow-up period (Table 2).

**Table 2:** Trial measures processes chart.

Items	Baseline measurement (week –2–0)	Intervention period (week 1–8)	Intervention outcome assessment (week 9)	Follow up period (week 9–12)	Follow up outcome assessment (week 12)	Analysis
Inclusion criteria	^∗^					^∗^
Exclusion criteria	^∗^					^∗^
Informed consent	^∗^					
Demographic information	^∗^					^∗^
Sas	^∗^		^∗^		^∗^	^∗^
Randomnization and allocation	^∗^					
Bioelectrical activity of cortical neurons	^∗^	^∗^	^∗^			^∗^
Electrical acticity of the heart	^∗^	^∗^	^∗^			^∗^
Self evaluation of qigong training		^∗^	^∗^			^∗^
Self report		^∗^	^∗^	^∗^	^∗^	^∗^
Adverse events		^∗^	^∗^	^∗^	^∗^	^∗^

The secondary outcome measures central nervous system EEG and cardiovascular ECG before and after qigong exercises in both sections. EEG and ECG data will be recorded at every visit (Table 3). The room temperature and humidity were controlled, and the testing protocol was designed to minimize fatigue and patient burden and maximize the validity of the data collected.

**Table 3:** Summary of outcome measures and associated variables.

	Physiological measurements	Testing methods	Outcome variable
Temporal dynamics during steady-state conditions	Heart rate	Beat-to-beat variation measured using ECG for a 10 minute during standing relaxing state	HR complexity CPC analysis
	EEG	Using EEG for a 10 minute during standing relaxing state, collect simultaneously with ECG	EEG complexity
Qigong dynamics during Three-circle post standing-state conditions	Heart rate	Beat-to-beat variation measured using ECG for a 10 minute during standing relaxing state	HR complexity CPC analysis
	EEG	Using EEG for a 10 minute during standing relaxing state, collect simultaneously with ECG	EEG complexity
Protocol adherence	Qigong training	Teachers report class attendance and records. Self-reported practice feelings and problems.	Hours of practice/week

The nervous system function will be collected using a 37-electrode EEG cap with an international 10 to 20 system with a linked mastoid reference (NeuroScan Co. USA.), 1000 Hz sampling rate, high pass filter of 0.05 Hz, low pass filter of 70 Hz, notch filter of 50 Hz, and impedance below 3 kΩ. We recorded 37 electrodes were recorded. The signals from these 37 electrodes were referenced to the linked mastoid lobe electrodes. All recordings were artifact-free EEG segments of 60 seconds duration. At the data pre-processing stage, independent component analysis was used to eliminate artifacts (eyes, muscle, and cardiac overlapping of the cardiac pulsation). Steady-state EEG dynamics are assessed during relaxation standing and will be collected for 5 minutes. Additionally, a trained research assistant will measure the EEG signal at the same time as the subject in the three-circle post standing position.

The heart rate (HR) was measured using MP-150 (BIOPAC Co. USA.), 1000 Hz sampling rate, and allowed a 5 minutes warm-up before the test. To collect a sufficient number of heartbeat cycles for complexity measures, HR dynamics were also assessed for 5 minutes simultaneously with EEG at both steady-state and three-circle post-standing states. Results in HR, with changes in HR response reflective of cardiovascular regulation, will be collected.

The breath rhythm will be calculated from ECG signals by the cardiopulmonary coupling method, and the breath method, depth, and frequency will be analyzed to compare the effects of breath regulation.

Subjects will be instructed to wear loose and comfortable clothing and shoes. The investigators will refrain from wearing laboratory coats during all sessions, as the use of such apparel has been associated with increased heart rate in some patients (white-coat hypertension).

### 2.18 Data analysis

The collected data will be recorded in standardized forms. The investigators will be supervised and verified when analyses are performed to avoid errors and minimize biases in the process.

Descriptive statistics were calculated for the dependent and independent variables. This analysis will include summary statistics of demographic information and SAS outcome measures. The SAS scores and demographic characteristics of subjects in the TCPSQ-BR and TCPSQ groups will be compared upon admission using a 2-sample *t*-test (continuous data). To control for baseline differences between the 2 groups, variables that were significantly different at baseline will serve as covariates in the analyses. Data will be analyzed on an intention-to-treat basis. IBM SPSS Statistics (version 25.0; SPSS Inc., Chicago, Illinois, United States) was used for all statistical analyses.

The EEG and ECG signals were exported in the European Data Format and processed using MATLAB R2018b (Mathworks Inc., Natick, MA) software was used for statistical evaluations. A prepost analysis of the heart rate variability (HRV) will be made, the complexity of heart rate and brain dynamics will be compared, and the coordination between mind and body will be measured by Granger causal decomposition.

Statistical analysis of each segment was applied. For each segment, the cross-sectional analysis between the 2 groups (TCPSQ-BR and TCPSQ) was assessed using one-way analysis of variance, as both normality and homogeneity of variance were not violated. Our analysis is based on a longitudinal regression analysis in which we do not have an estimate of the within-participant correlations. Nonparametric methods are used when assumptions of normality are violated. The alpha level was set at *P* < .05.

Adverse events will be reported to the data and safety monitoring board. Adverse events will be recorded when participants present with complaints at any time and will be based on safety policies and adverse event forms.

Serious adverse events will be recorded as required by the International Conference on Harmonization Guidelines E2A. We will submit the results of the trial for publication in an appropriate journal, irrespective of the outcome. We report the trial in accordance with CONSORT statements.

## 3 Discussion

Our goal was to determine the anxiety-release effect of qigong practice, and to analyze the possible mechanism by comparing the change in the timeline in the synergy of EEG and HRV before and after TCPSQ practice between the 2 groups. Here, we hypothesize that anxiety decreases after TCPSQ practice because the mind and body are relaxed and balanced, and the complexity system functions are associated with each other. We will compare the difference between anxiety subjects and healthy subjects’ bioelectrical signal changes to investigate the mechanism of qigong's anxiety-release effects. We will analyze the connections between complexity measures and functional capacity at baseline and 12 weeks later, and investigate whether changes in EEG are associated with changes in HRV. We will not adjust for multiple testing in this study, and we will include all available data.^[49]^ Our primary analysis employs a nonlinear metric of complexity measure that examines changes over time for each outcome measure and for each of the systems. Therefore, the primary endpoint for this study was the change in EEG, HRV, and their synergistic effects from baseline to 12 weeks posttherapy. The primary comparison was between participants randomly assigned to TCPSQ-BR practice versus TCPSQ practice only. Subgroup analyses for anxiety, age, and gender were conducted by including each classification. Mixed effects models will be used to examine the effects of Qigong practice over time on physical and mental function, as well as changes in heart rate and brain dynamics.

As one of the representative mindful exercises, CMQ is a famous mind-body aerobic practice that originated from the Traditional Chinese Medicine,^[32]^ and has been found to have effects on various conditions, such as preventing and curing psychological and physical diseases. CMQ is characterized by initiative exercises to regulate body, breath, and mind and to integrate them into 1. It has been widely used clinically because of its relaxing and healing effects. Many qigong researchers consider that the qigong effects on the human body are systemic and not only work on the body, but also on the mind. However, the mechanisms underlying these 2 systems and how they relate to each other remain unclear. As an initiative exercise, Qigong practice was performed by the practitioner themselves, which means qigong is a consciousness-led practice. This is why when practitioners reach a state of oneness with three adjustments, the functions of the body and brain could be regulated simultaneously. A number of questions we facing is that limit of our ability to evaluate complexity-based physiological metrics as biomarkers for monitoring complex system, as well as their value for quantifying the impact of mindful exercises such as Qigong on mind body regulation and their interaction. Through a Granger causal decomposition analysis for complexity and systematic physiological signals, this study will characterize qigong's impact on moment-to-moment variations in dynamic systems, as well as the relationship between physical and mental function.

Physiologic complexity is typically estimated using a number of techniques derived from the fields of nonlinear dynamics and statistical physics that quantify the moment-to-moment quality, scaling, and/or correlation properties of dynamic signals^[54]^ Compared to other entropy-based methods, MSE is uniquely representative for encoding dynamical information in physiological signals over multiple scales, and is able to distinguish between highly irregular or uncorrelated complex physical signals. MSE has the advantage of measuring the irregularity time series under different time scales.^[55]^ It is designed to reflect the degree of health condition of a biological system by its output physiological signals,^[56]^ which is particularly developed to analyze nonlinear and non-stationary constant signals and their relationship. It has been hypothesized that as part of complexity physiologic systems, EEG and HRV have similar changing trends on the same time scale.^[43]^ However, experimental evidence supporting this hypothesis is limited. In this study, we evaluated whether the widely utilized complexity-based metrics (MSE) are sensitive and informative metrics for characterizing the impact of qigong on complexity in cardiovascular and nervous systems.^[57–59]^

Aforementioned we briefly summarized our studies protocol that support the value of complexity-based biomarkers in characterizing correlation in cardiovascular and nervous dynamics. This study will prove its mind body effects by to test the methods for future randomized trials to investigate further the clinical value of qigong in mind body diseases.

## Author contributions

**Conceptualization:** Jiaxuan Lyu, Yulong Wei.

**Data curation:** Jingjing Dong, Hangyu Li, Xinzheng Zhang.

**Investigation:** Jiaxuan Lyu.

**Methodology:** Jiaxuan Lyu, Yulong Wei.

**Project administration:** Jiaxuan Lyu, Yulong Wei.

**Supervision:** Jiaxuan Lyu, Yulong Wei.

**Validation:** Jiaxuan Lyu.

**Visualization:** Jiaxuan Lyu, Jingjing Dong, Hangyu Li, Xinzheng Zhang.

**Writing – original draft:** Jiaxuan Lyu.

**Writing – review & editing:** Yulong Wei.
